# Increased Fibrosis in a Mouse Model of Anti-Laminin 332 Mucous Membrane Pemphigoid Remains Unaltered by Inhibition of Aldehyde Dehydrogenase

**DOI:** 10.3389/fimmu.2021.812627

**Published:** 2022-02-07

**Authors:** Sabrina Patzelt, Manuela Pigors, Heiko Steenbock, Leonard Diel, Katharina Boch, Lenche Chakievska, Sven Künzel, Hauke Busch, Anke Fähnrich, Jürgen Brinckmann, Enno Schmidt

**Affiliations:** ^1^Lübeck Institute of Experimental Dermatology, University of Lübeck, Lübeck, Germany; ^2^Institute of Virology and Cell Biology, University of Lübeck, Lübeck, Germany; ^3^Department of Dermatology, Allergology and Venerology, University of Lübeck, Lübeck, Germany; ^4^Max Planck Institute for Evolutionary Biology, Plön, Germany; ^5^Institute for Cardiogenetics, University of Lübeck, Lübeck, Germany

**Keywords:** aldehyde dehydrogenase, autoimmune blistering disease, collagen, crosslinking, fibrosis, laminin 332, mouse model, mucous membrane pemphigoid

## Abstract

Mucous membrane pemphigoid (MMP) is an autoimmune blistering disease characterized by autoantibodies against the basal membrane zone of skin and surface-close epithelia and predominant mucosal lesions. The oral cavity and conjunctivae are most frequently affected, albeit clinical manifestations can also occur on the skin. MMP-associated lesions outside the oral cavity typically lead to scarring. Mechanisms underlying scarring are largely unknown in MMP and effective treatment options are limited. Herein, we assessed the collagen architecture in tissue samples of an antibody-transfer mouse model of anti-laminin-332 MMP. In MMP mice, increased collagen fibril density was observed in skin and conjunctival lesions compared to mice injected with normal rabbit IgG. The extracellular matrix of MMP skin samples also showed altered post-translational collagen cross-linking with increased levels of both lysine- and hydroxylysine-derived collagen crosslinks supporting the fibrotic phenotype in experimental MMP compared to control animals. In addition, we evaluated a potential anti-fibrotic therapy in experimental anti-laminin-332 MMP using disulfiram, an inhibitor of the aldehyde dehydrogenase (ALDH), which has been implicated in immune-mediated mucosal scarring. In addition, disulfiram also acts as a copper chelator that was shown to block lysyl oxidase activity, an enzyme involved in formation of collagen crosslinks. Topical use of disulfiram (300 μM in 2% [w/v] methocel) did not improve ocular lesions in experimental MMP over the 12-day treatment period in disulfiram-treated mice compared to vehicle-treated mice (n=8/group). Furthermore, C57BL6/J mice (n=8/group) were treated prophylactically with 200 mg/kg p.o. disulfiram or the solvent once daily over a period of 12 days. Systemic treatment did not show any reduction in the severity of oral and ocular lesions in MMP mice, albeit some improvement in skin lesions was observed in disulfiram- vs. vehicle-treated mice (p=0.052). No reduction in fibrosis was seen, as assessed by immunohistochemistry. Whilst blocking of ALDH failed to significantly ameliorate disease activity, our data provide new insight into fibrotic processes highlighting changes in the collagenous matrix and cross-linking patterns in IgG-mediated MMP.

## Introduction

Mucous membrane pemphigoid (MMP) is an autoimmune blistering disease characterized by autoantibodies against structural proteins of the basal membrane zone of skin and surface-close epithelia and predominant mucosal lesions (1, 2). Most frequently, the oral cavity and conjunctivae are affected, in 20% of patients also the skin (3, 4). MMP lesions in the nose, pharynx, larynx, trachea, esophagus, skin, and particularly, in the conjunctivae, heal with scarring leading to complications, such as laryngeal, tracheal and esophageal narrowing and impaired vision that may proceed to blindness (1, 5). The C-terminus of BP180 (collagen XVII) and laminin-332 represent two of the critical targets in the scarring phenotype of MMP (2). Both molecules extend from the lamina lucida into the lamina densa of the basement membrane zone (6, 7), and a disruption caused by autoantibodies directed against the BP180 C-terminal stretch as well as laminin-332 triggers an inflammatory cascade and immune cells, such as macrophages, release a number of profibrotic cytokines (e.g. transforming growth factor β, interleukin-13), which in turn activate fibroblasts and initiate the fibrosis response (3, 5, 8–12).

A previously established mouse model of MMP, induced by antibody transfer of IgG directed against the laminin alpha 3 chain (Lama3), mimicked characteristic clinical and immunopathological features of the human disease including linear deposits of IgG and C3 at the basal membrane zone of skin and surface-close epithelia, subepithelial blistering, and erosions or blisters in the oral cavity, conjunctivae, esophagus, and skin (13). Animal models of MMP and other pemphigoid diseases, including, bullous pemphigoid and epidermolysis bullosa acquisita, have provided mechanistic insight in disease pathways underling the inflammatory processes and blister formation in autoantibody-induced tissue injury (14–16), yet, the mechanisms underlying scarring fibrosis have not been investigated in these experimental models.

Current therapies of MMP are aimed at managing inflammation and as such only indirectly prevent progressive fibrosis. Therapeutic regimens mainly rely on the long-term use of high-dose corticosteroids in combination with potentially corticosteroid-sparing agents such as azathioprine, mycophenolate mofetil, cyclophosphamide, or intravenous immunoglobulin (2, 17). With the aim of identifying more effective therapy options for mucocutaneous autoimmune diseases, particularly for the severe consequences of tissue scarring, Ahadome et al. previously demonstrated that aldehyde dehydrogenase family 1 (ALDH1) is implicated in ocular mucosal scarring and inflammation (18). ALDH1 was shown to be upregulated in conjunctiva and fibroblasts of ocular MMP patients. Conversely, ALDH inhibition with disulfiram, which can block both cytosolic ALDH1 and mitochondrial ALDH2 isoforms (19), led to a decrease in fibrosis in a mouse model of scarring allergic eye disease.

Here, we evaluated the anti-fibrotic and anti-inflammatory properties of the ALDH inhibitor disulfiram in the antibody-transfer induced mouse model of anti-laminin-332 MMP. Whilst our data provide evidence for fibrotic changes and dysfunctional collagen-crosslinking in MMP skin, ALDH inhibition did not significantly ameliorate skin and mucosal lesions in experimental MMP.

## Materials And Methods

### Mice

All animal experiments were approved by the Schleswig-Holstein Ministry of Energy Transition, Agriculture, Environment, Nature and Digitalization (40-3/15, 20-2/17). Adult C57BL/6J mice were purchased from Charles River (Wilmington, MA, USA). Mice were housed at the animal facility of the University of Lübeck under sterile conditions (SPF, specific pathogen-free) with a 12-hour light-dark cycle and free access to food and water. Clinical examinations were performed on anesthetized mice. Anesthesia was initiated with i.p. administration of ketamine (100 µg/g) and xylazine (15 µg/g). Mice were sacrificed under anesthesia by cervical dislocation and tissue biopsies were taken for subsequent analyses.

### Antibody-Transfer Mouse Model of MMP

The induction of experimental MMP has been described in detail previously (13, 20). In brief, anti-murine laminin alpha 3 (mLama3) IgG was generated by immunization of New Zealand white rabbits with recombinant His-tagged fragments directed against the mid (amino acids 1656–1985) and C-terminal (amino acids 2756–3330) domains of mLama3. Total rabbit IgG was affinity purified using protein G Sepharose (Genscript, Piscataway, NJ, USA). C57BL/6J mice were injected s.c with 6 mg anti-mLama3 IgG or normal rabbit IgG every other day from Day 0 to Day 10 to induce disease. Experimental groups were distributed in a blinded manner and mixed within the cages.

### Treatment

For local treatment of ocular lesions, disulfiram (Tetraethylthiuram disulfide, Sigma-Aldrich, Munich, Germany) was dissolved to a concentration of 300 μM in 2% (w/v) methocel ([Hydroxypropyl-methylcellulose], Sigma-Aldrich, Munich, Germany) and administered to the diseased C57BL/6J mice (n=8) daily from Day 8 to Day 19 as eye drops. The control group (n=8) received methocel only. In addition, C57BL/6J mice (n=8/group) were treated systemically with p.o. 200 mg/kg disulfiram once daily or olive oil (ALDI, Essen, Germany) over a period of 12 days.

### Scoring of Mice

The extent of affected body surface area was determined during anesthesia and was done in a blinded fashion every fourth day as described (13, 21). For evaluation of eye lesions, the percentage of affected ocular surface, with each eye contributing to 50% to the total percentage of the affected eye area, was determined. Swellings, crusts, and erosions were rated equally, regardless of their extent or severity.

On the last day of the experiments, mice were narcotized and endoscopy of the oral cavity was performed (HOPKINS Optik 64019BA; Karl StorzAidaVet, Tuttlingen, Germany) to assess the extent of oral erosions and blisters present on the tongue, left/right cheek, and pharyngeal mucosa. The presence of lesions, on one of these sites results in a score of 1; if lesions on all four sites occur, the maximum score of 4 is given.

To quantify the severity of conjunctival lesions in the mice, biopsies of the palpebral conjunctiva were taken on the last day of the experiment, preserved in 4% Histofix Solution (Carl Roth, Karlsruhe, Germany) and embedded in paraffin. Sections with a thickness of 5 µm were cut from three different areas within the tissue block (100 micrometers apart) and stained with hematoxylin & eosin according to the standard protocols. Of these, only the sections showing the largest split at the level of the basement membrane zone were evaluated, whereby the following criteria had to be met: (i) The conjunctiva has a length of at least 1000 µm. (ii) The split is not at the end of the section. The length of the split formation in the conjunctiva was determined using Keyence BZ-II Analyzer software (vs 2.1) and scored as follows: no split formation: score = 0; split 1–100 µm: score = 1; split 101–200 µm: score = 2; split 201–300 µm: score = 3; split ≥ 300 µm: score = 4.

### Histological Stainings

For the Picrosirius red staining, a Picrosirius red solution was prepared by dissolving 0.5 mg of Direct Red 80 Dye (Sigma-Aldrich, Munich, Germany) in 500 ml of saturated 1.2% picric acid (Fisher Scientific, Schwerte, Germany). After deparaffinization and rehydration, 5 µm-thick paraffin sections were stained with Mayer’s Haematoxylin (Waldeck, Münster, Germany) for 10 minutes. The slides were then rinsed in running tap water for 10 minutes and counter-stained with picrosirius red solution for 1 hour. The slides were briefly rinsed in distilled water and washed in two changes of acidified water for 2x 5 minutes. The water from the slides was removed by vigorous shaking, dehydrated through two changes of 100% ethanol for 2x 5 minutes, and two changes of xylene for 2x 5 minutes, and cover-slipped with resinous mounting medium. The sections were imaged using a Keyence microscope (BZ-9000 series, Keyence GmbH, Neu-Isenburg, Germany). Images were captured using identical settings and exposure time. Quantification of collagen fibril density was performed using ImageJ (version 1.53i, https://imagej.nih.gov/ij/). All images were converted to gray scale and red-stained collagen isolated using the same thresholding for all images. The mean percentage of Picrosirius red positive area (± SD) was calculated from two to five images from each biopsy.

The Masson-Goldner’s trichrome staining was performed as previously described (22).

### Indirect Immunofluorescence Staining

Indirect immunofluorescence staining was performed as described (23, 24). Antibodies included Alexa Fluor 594 anti-mouse F4/80 clone BM8 and Alexa Fluor 594 anti-mouse CD11c clone N418 (both Biolegend, San Diego, California, USA) as well as DAPI (Roche Diagnostics, Mannheim, Germany).

### Protein and Collagen Crosslink Analysis

4-mm punch biopsies of lesional skin from the head/neck area and conjunctiva as well as 1 cm of apical esophagus were obtained from diseased and non-diseased female C57BL/6J mice (n=6/group) on Day 28 and stored at -20°C prior to processing. For crosslink analysis, samples (about 20 mg wet weight) were reduced by sodium borohydride (Sigma-Aldrich, Munich, Germany; 25 mg NaBH_4_/ml in 0.05 M NaH_2_PO_4_/0.15 M NaCl pH 7.4, 1 h on ice, 1.5 h at room temperature) to stabilize acid-labile collagen crosslinks. Specimens were digested with high purity bacterial collagenase (C0773; Sigma-Aldrich, Munich, Germany; two times 50 U/ml, 37°C, 18 h). After centrifugation, the soluble fractions (collagen crosslinks) were hydrolyzed in 6 N HCl at 110°C for 24 h. The hydrolyzates were pre-cleared by solid-phase extraction to remove the bulk of non-crosslinked amino acids (Agilent, USA). Dried eluates were re-dissolved in sodium citrate loading buffer (pH 2.2) and analyzed on an amino acid analyzer (Biochrom 30, Biochrom, Cambridge, UK) using a three-buffer gradient system and post column ninhydrin derivatization. The column was eluted for 5 min (flow rate 15 ml/h) with sodium citrate buffer (pH 4.25), for 40 min with sodium citrate buffer (pH 5.35) and for 20 min with sodium citrate/borate buffer (pH 8.6) at 80° C. Retention times of individual crosslinks were established with authentic cross-link compounds. Quantitation was based on ninhydrin generated leucine equivalence factors (dihydroxylysinonorleucine [DHLNL], hydroxylysinonorleucine [HLNL]: 1.8; hydroxyllysylpyridinoline [HP]: 1.7) (25). The nomenclature used in the manuscript refers to the reduced variants of cross-links (DHLNL, HLNL). The collagen and the non-collagenous protein content of the specimens were analyzed in an aliquot of hydrolyzed samples of the soluble and the residual fractions prior to solid phase preclearance. Collagen content was calculated based on a content of 14 mg hydroxyproline in 100 mg collagen.

### Transcriptome Analysis

Perilesional skin biopsies were obtained from mice after repeated injection of anti-mLama3 IgG adult (n=5) or normal rabbit IgG (n=3). Tissue samples were taken on the last day of the experiment (Day 12) and stored in RNAlater (Qiagen, Hilden, Germany). Processing of RNA and subsequent next-generation sequencing was carried as described previously (26). Fastq reads were pseudo-aligned to the GRCm38 mm10 genome assembly using Kallisto (27) and transcript read counts were aggregated to Ensembl Gene IDs for further analysis. Differential gene expression analysis was performed *via* the R library sleuth (28) using a linear model that accounted for batch effects. Significance and effect sizes of differential gene regulation were calculated from the likelihood ratio and the Wald test, respectively, as implemented in the sleuth package.

### Statistical Analysis

GraphPad Prism (Version 7, GraphPad Software, San Diego, CA, USA) was used for statistical analysis. All data presented as mean ± standard deviation. Unless otherwise stated, an unpaired t-test was performed to determine significance. For all analyses, *p* < 0.05 was considered statistically significant.

## Results

### Increased Collagen Fibril Density and Altered Post-Translational Collagen Cross-Linking Contribute to Fibrotic Changes in Experimental MMP

**Figure 1:**
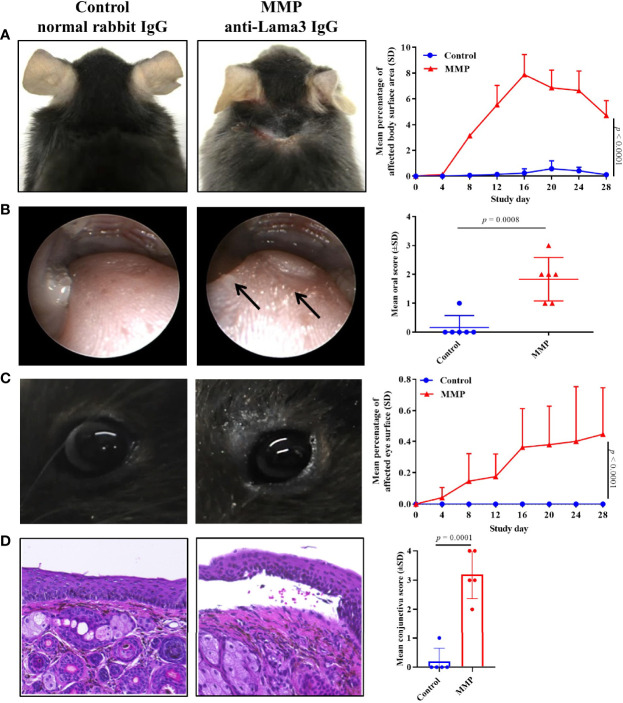
The mouse model of mucous membrane pemphigoid (MMP) recapitulated phenotypic features of human disease up to four weeks. **(A)** Throughout the 28-day study, injection of anti-laminin alpha 3 (Lama3) IgG led to development of crusts and erosions on the skin mainly presenting on the head and neck area (n=8/group). One mouse/group died during the course of the study. **(B)** Endoscopy showed prominent oral lesions in MMP mice on Day 28. **(C)** Anti-Lama3 IgG-injected mice presented with periocular crusted erosions and conjunctival inflammation. **(D)** Consistent with the presence of ocular lesions, histological analysis showed subepithelial cleavage and leukocyte infiltrates in palpebral conjunctiva biopsies of MMP mice **(D)**. Mice injected with normal rabbit IgG served as a control and did not show any meaningful clinical or histological manifestations. A two-way ANOVA with Sidak’s multiple comparisons test was used to calculate significance **(A, C)**.

Experimental MMP was induced in C57BL/6J mice by transfer of rabbit anti-mLama3 IgG directed at recombinant fragments of mid and C-terminal domains, as described (13). Anti-mLama3 IgG was injected at a dose of 6 mg/mouse/injection subcutaneously into the neck of C57BL/6J mice (n = 6) every second day from Day 0 to Day 10. Control mice received the comparable dose of normal rabbit IgG. In line with our previous report, mice treated with anti-mLama3 IgG developed erosions and crusts primarily on the head and neck, forelegs, around the ears, eyes, and snout that were present up to Day 28 with a mean (± SD) affected body surface area of 4.71 (±1.16) (**Figure 1A**). Skin histology showed subepithelial split formation and a dense infiltrate of inflammatory cells particularly in the upper dermis (**Supplemental Figure 1**). In addition, mice developed severe lesions in the oral cavity as assessed by endoscopy, with erosions and blisters present on the tongue, oral mucosa, and pharynx (**Figure 1B**). The oral severity disease score integrates affected areas on the buccal (left/right), tongue, and pharyngeal mucosa, as described in the Materials & Methods section. Ocular lesions present in MMP mice were confirmed on a histological level by presence of subepithelial split formation accompanied by leukocyte infiltration below the basement membrane (**Figures 1C, D****)**. Control mice challenged with normal rabbit IgG did not develop any clinical or histological alterations (**Figures 1A–D**).

**Figure 2:**
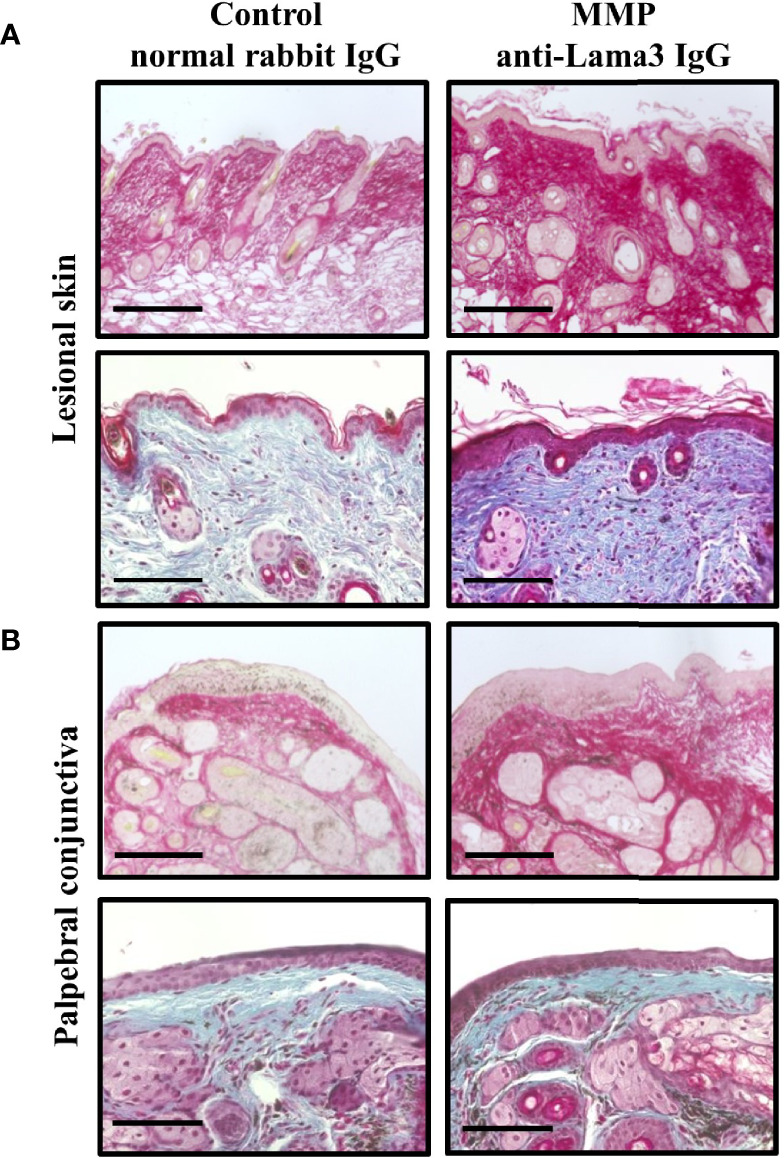
Experimental mucous membrane pemphigoid (MMP) showed an increased collagen fibril density. **(A, B)** MMP mice injected with anti-laminin alpha 3 (Lama3) IgG showed increased collagen density in lesional skin **(A)** and palpebral conjunctiva **(B)** on Day 28, as assessed by Picrosirius red staining (top row of panels A and B) and Masson’s trichrome staining (bottom row of panels A and B). No increases in collagen fibril density were seen in control mice treated with normal rabbit IgG. Scale bars = 100 µm.

Pemphigoid animal models were shown to recapitulate distinct phenotypic and immunopathological features of the different disease subtypes, yet scarring fibrosis has not been investigated *in-vivo*. To assess fibrotic changes in the anti-mLama3 IgG-induced MMP mouse model we first analyzed the collagen architecture in murine tissue samples obtained on Day 28. Picrosirius red staining, which stains collagens with a deep red color under a brightfield microscope, showed increased collagen fibril density in tissue samples from skin and palpebral conjunctiva in MMP compared to healthy control mice treated with normal rabbit IgG (**Figures 2A, B****)**. In addition, Masson’s trichrome staining, which shows collagen-rich fibrotic regions in blue, confirmed the presence of increased collagen fibril thickness in MMP tissue samples (**Figures 2A, B****)**.

**Figure 3:**
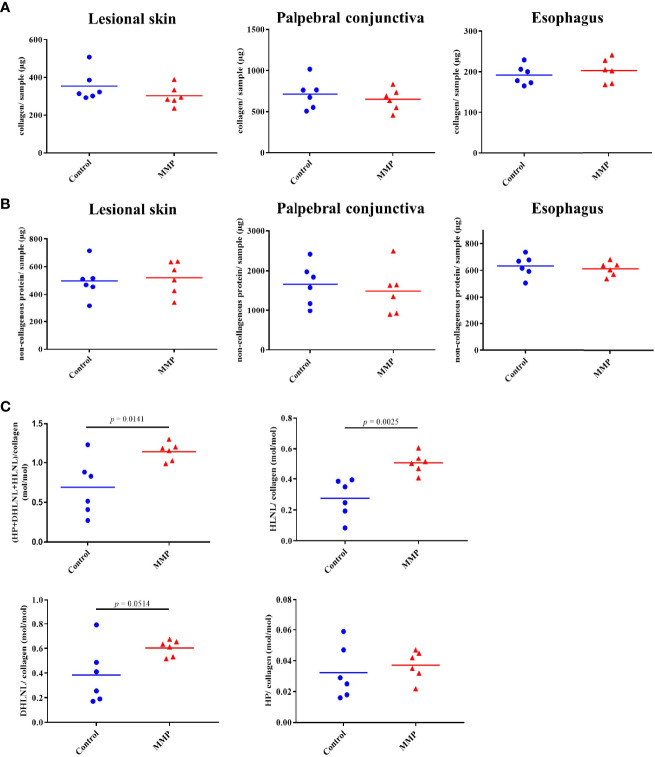
Experimental mucous membrane pemphigoid (MMP) showed elevated levels of collagen crosslinks. **(A, B)** Amino acid analysis showed no changes in collagen content **(A)** as well as non-collagenous proteins **(B)** in MMP vs. control mice. **(C)** Significant increases in total collagen cross-links as well as HLNL were observed in MMP lesional skin samples, compared with healthy control mice. In addition, higher levels of DHLNL were measured in MMP lesional skin, whereas levels of the mature cross-link HP were comparable in both groups. HP, Hydroxyllysylpyridinoline; DHLNL, dihydroxylysinonorleucine; HLNL, hydroxylysinonorleucine.

Next, we performed biochemical analysis of defined tissue biopsies obtained from lesional skin, conjunctiva, and esophagus to quantify collagen and non-collagenous proteins as well as different components of the collagen cross-linking pathways. In contrast to the histopathological findings, no increases in either collagen or non-collagenous proteins were observed (**Figures 3A, B****)**. Nevertheless, amino acid analysis showed changes in different components of two main collagen cross-link pathways, namely the allysine and hydroxyallysine route (29, 30). Lysine aldehyde-derived collagen crosslinks (allysine pathway), like hydroxylysinonorleucine (HLNL), are characteristic for soft tissues, such as the skin. Hydroxylysine aldehyde-derived collagen cross-links (hydroxyallysine pathway) include the divalent crosslink dihydroxylysinonorleucine (DHLNL) and its maturation product hydroxylysylpyridinoline (HP) and are typically observed in hard tissues, such as bone and cartilage. Increases in these crosslinks are also associated with fibrosis (31–33). Significant increases in HLNL and total crosslinks were observed in lesional skin of MMP mice compared with the control group (**Figure 3C**). Increased levels of DHLNL were also observed in lesional skin of diseased mice, however, these changes were not statistically significant. No meaningful changes in HP were detected in MMP skin biopsies (**Figure 3C**). Collagen crosslinks in tissue biopsies from conjunctiva and esophagus were unchanged (data not shown).

In light of a potential new therapeutic approach using the ALDH inhibitor disulfiram, we also assessed transcriptome data from an independent study, where perilesional skin was obtained from mice with anti-mLama3 IgG-induced MMP at Day 12. In this data set, we specifically searched for significantly upregulated RNA levels of *ALDH* isoforms, as Ahadome et al. previously demonstrated that particularly ALDH1 isoforms contribute to fibrotic scarring in ocular MMP (18). In line with this report, we also detected significantly increased levels of *Aldh1b1* as well as *Aldh3b1, Aldh16a1*, and *Aldh18a1* demonstrating that other ALDH isoforms potentially also contribute to fibrotic scarring in MMP (**Table 1**).

**Table 1:** Upregulated *ALDH* isoforms in perilesional skin of MMP mice.

ALDH isoform	Transcript identifier	*p-*value	Effect size
*Aldh16a1*	ENSMUSG00000007833	0.0332	0.3758
*Aldh18a1*	ENSMUSG00000025007	0.0607	0.3212
*Aldh1b1*	ENSMUSG00000035561	0.0468	0.6140
*Aldh3b1*	ENSMUSG00000024885	0.0180	0.5280

### Topical Application of Disulfiram Did Not Ameliorate Ocular Lesions in Experimental MMP

**Figure 4:**
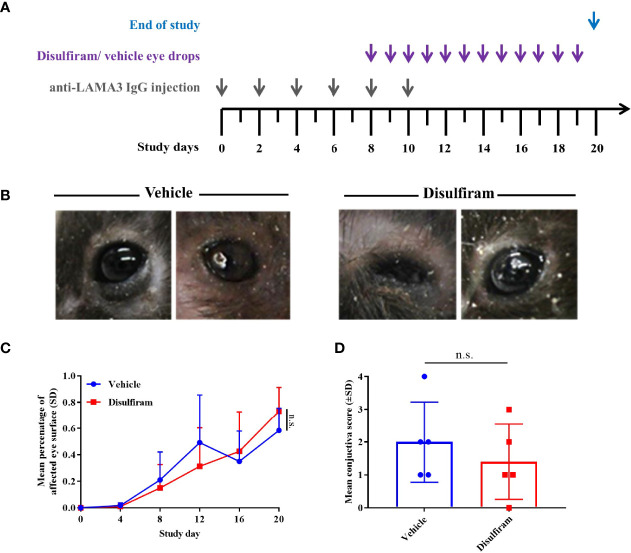
Local use of the ALDH inhibitor disulfiram did not improve conjunctival lesions in experimental MMP. **(A)** Disulfiram or the respective solvent were administered to the diseased mice (n = 8/group) daily from Day 8 to Day 20 as eye drops. **(B)** Mice with experimental MMP presented with progressive conjunctival scarring and corneal changes on Day 20. Statistical analysis was performed using two-way ANOVA with Sidak’s multiple comparisons test. **(C)** On Day 8, the mice had an average affected ocular surface area of approximately 20%. Over the treatment period of 12 days, no improvement in the extent of ocular lesions was observed as a result of the topical treatment in both groups. **(D)** No significant difference in conjunctiva scores was present in vehicle- vs. disulfiram-treated tissue biopsies. n.s., not significant.

Since signs of fibrosis were present in the antibody-transfer mouse model of MMP, we aimed at evaluating the long-known ALDH inhibitor disulfiram, which has previously been proposed as a promising new anti-inflammatory and anti-fibrotic treatment option in ovalbumin-induced allergic scarring conjunctivitis (18). In addition, disulfiram was also shown to block activity of lysyl oxidases, which aid in catalyzing the cross-linking reaction of collagens (34). Disulfiram was dissolved to a concentration of 300 μM in 2% (w/v) methocel and administered to the diseased mice (n = 8/group) daily from Day 8 to Day 19 in the form of eye drops (**Figure 4A**). Upon start of the treatment on Day 8, the mice had a mean affected eye surface area of 21% and 15% in the vehicle vs. disulfiram group. On Day 20, mice of both the vehicle and disulfiram groups presented with severe ocular lesions characterized by crusted erosions and progressive conjunctival scarring respectively (**Figure 4B**). No improvement in the extent and severity of eye lesions was observed as a result of the topical treatment in both treatment and control groups over the treatment period of 12 days and vehicle vs. disulfiram-treated mice showed no significant differences in the mean affected eye surface area on Day 20 (58% vs. 73%) (**Figure 4C**). In addition, histological quantification of subepithelial split formation (conjunctiva score) showed no difference between the treatment and control group (**Figure 4D**).

### Systemic Treatment With the ALDH Inhibitor Disulfiram Did Not Resolve Clinical Lesions in Experimental MMP

**Figure 5:**
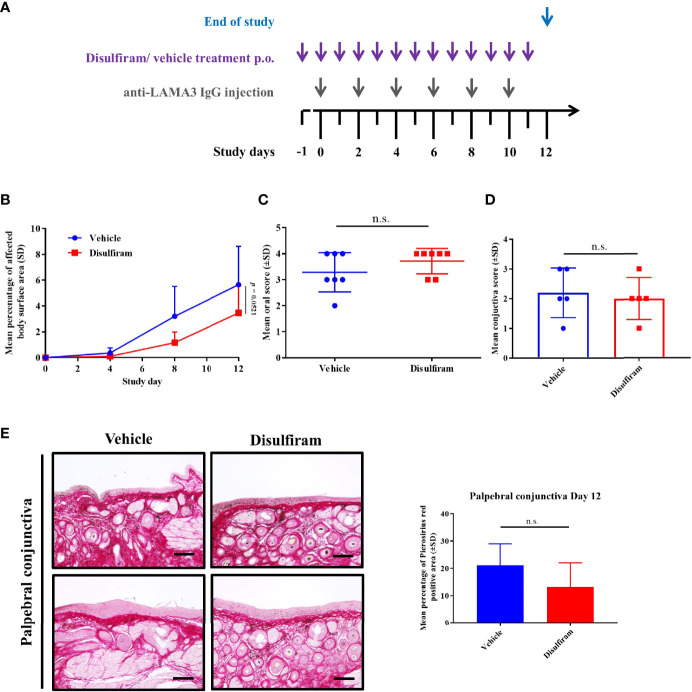
Systemic treatment with the ALDH inhibitor disulfiram did not resolve skin and mucosal lesions in experimental MMP. **(A)** C57BL6/J mice (n = 8/group) were treated prophylactically with p.o. 200 mg/kg disulfiram once daily or the respective solvent, i.e. corn oil, over a period of 12 days. **(B)** No significant changes in affected body surface area were observed in both treatment groups, though disulfiram-treated mice showed a trend towards improvement in clinical skin scores (p = 0.0521). Statistical analysis was performed using two-way ANOVA with Tukey’s multiple comparisons test. **(C, D)** In addition, endoscopy and histological analysis showed no improvement in the severity of oral mucosal lesions **(C)** or conjunctiva scores **(D)**, respectively, in vehicle- vs. disulfiram-treated mice. **(E)** No significant reduction in collagen fibril density was seen in vehicle vs. disulfiram-treated mice, as assessed by Picrosirius red staining. Scale bar = 50 µm. n.s., not significant; SD, standard deviation.

Since topical treatment did not show any meaningful therapeutic effects in experimental MMP, we assessed whether systemic use of disulfiram would improve MMP-associated lesions in mice. C57BL6/J mice (n = 8/group) were injected s.c. with anti-mLama3 IgG every other day over a period of 10 days to induce experimental MMP (**Figure 5A**). In parallel, mice were treated prophylactically, starting one day before the induction of the disease, with p.o. 200 mg/kg disulfiram once daily or the respective solvent, i.e., olive oil, over a period of 12 days. One mouse of each group died during the study. No significant changes in affected body surface area were observed in both treatment groups, though disulfiram-treated mice showed a trend towards improvements in clinical skin scores (*p* = 0.0521) (**Figure 5B**). Skin histological findings with subepidermal spilt formation and inflammatory infiltrate, including macrophages (F4/80) as well as dendritic cells (Cd11c), were similar between vehicle and disulfiram-treated MMP mice (**Supplemental Figure 2**). Endoscopy was performed at Day 12 to determine the extent of oral involvement. No difference in the extent of oral lesions was observed in the disulfiram-treated mice compared to vehicle-treated animals (**Figure 5C**). In line with the lack of topical treatment response, no improvement in the extent and severity of ocular lesions was seen (**Figure 5D**), and no reduction in fibrosis was apparent on a histological level, as demonstrated by comparable density in collagen fibrils in control vs. disulfiram-treated mice (**Figure 5E**).

## Discussion

Consistent with the fibrotic changes observed in experimental MMP, fibrotic scarring is commonly seen in chronic or recurrent lesions of skin and mucous membrane in patients with MMP (2, 10, 35). In particular, scarring is of high clinical relevance in the conjunctivae due to the associated progressive visual impairment (5). Cutaneous lesions manifesting as tense blisters typically present on the scalp, face, neck, inguinal areas, and limbs. Typically, skin lesions in MMP heal with scarring and milia formation (36, 37). Scarring of the skin can result in discoloration and patchy areas of hair loss. This phenotype is often associated with the Brunsting-Perry pemphigoid subtype, which, predominantly presents with skin lesions on the head and neck healing with atrophic scarring (1, 36–38). Mucous membrane involvement does occur in patients with the Brunsting-Perry pemphigoid, albeit infrequently. Anti-fibrotic therapies, including those for cicatricial conjunctivitis in MMP, are scarce and of limited effect. The aim of the present study was, to determine the extent of fibrosis in a mouse model of MMP and to evaluate the anti-fibrotic properties of the ALDH inhibitor disulfiram.

Fibrotic scarring is typically marked by elevated levels of extracellular matrix proteins, particularly collagens, which provide mechanical integrity and resiliency to various tissues (39, 40). Besides the increased collagen deposition, changes in posttranslational cross-linking of collagen were demonstrated to contribute to increased tissue stiffness and fibrosis in various diseases associated with collagen abnormalities, including sclerotic skin conditions, such as systemic sclerosis, lipodermatosclerosis, keloids, and hypertrophic scars (31, 32, 41–43).

The formation of enzymatic collagen crosslinks is driven by lysyl oxidases, which catalyze the oxidative deamination of lysine or hydroxylysine residues in the telopeptides of collagens into allysine or hydroxyallysine aldehydes, respectively (29, 30, 44). These aldehydes subsequently react with helical lysine or hydroxylsyine residues leading to formation of divalent collagen crosslinks, e.g. HLNL (allysine pathway) or DHLNL (hydroxyallysine pathway), the latter of which is further processed into the trivalent crosslink HP (hydroxyallysine pathway) that contributes to the stiffness and strength of the functional collagen fibrils. HLNL can be formed between telopeptidyl allysine and helical hydroxylysine or telopeptidyl hydroxyallysine and helical lysine and, thus, belongs to both, the allysine or hydroxyallysine pathway. In rodent skin, the helical lysine residue utilized in HLNL is completely hydoxylated, so, here, HLNL belongs to the allysine pathway (45).

Here, we showed that in both lesional skin and palpebral conjunctiva, fibrosis was present in mice four weeks after induction of experimental MMP compared to mice injected with control IgG. In addition, immature collagen crosslinks of both the allysine and hydroxyallysine routes are elevated in MMP lesional mouse skin. Histological analysis using both Pircrosirus red and Masson’s trichrome staining showed an increased fibril density due to these changes in collagen crosslinks rather than an increased collagen deposition in the extracellular matrix. Similar observations were made for arthrosis, where bone tissue showed an increase in collagen density but not in collagen concentration (46). In healthy skin, allysine pathway-derived collagen crosslinks are typically prevalent. Elevated levels of soft tissue crosslinks in the MMP mouse samples are consistent with previous studies, where increases in lysine-derived crosslinks as well as allysine content were shown to be associated with systemic sclerosis and early stages of pulmonary fibrosis as a result of increased lysyl oxidase activity (47–50). Characteristic for pathologic fibrosis in soft tissues such as the skin, lung, liver, and kidney is also an increased formation of hydroxyallysine crosslinks including DHLNL and HP, which are typically seen in stiff connective tissues (31–33, 41, 42, 51–56). In line with these reports, we observed increases in the hydroxylysine-derived crosslink DHLNL in MMP skin. The marked accumulation of collagen crosslinks may contribute to increased tissue stiffness and progressive fibrotic scarring and particularly elevated levels of hydroxylysine-derived crosslinks were demonstrated to increase matrix stiffness due to a lower susceptibility to proteolytic degradation (54). Signs of fibrosis were also visible in the conjunctiva; however, these observations were not supported by changes in collagen crosslinks. Further analysis is required to assess fibrosis in conjunctiva tissue of MMP mice, as our analysis was limited to biopsies from the palpepbral conjunctiva on Day 28. Other parts of the ocular surface sheet, including bulbar conjunctiva and cornea, may show more pronounced fibrosis and altered cross-linking patterns. Taken together, the main feature of MMP-induced fibrosis on a biochemical level is an increase in total collagen crosslinks in lesional skin, which may be due to an increase in lysyl oxidase gene expression or enzyme activity. While this has not been documented for MMP yet, studies of injured human skin of recessive dystrophic epidermolysis bullosa patients showed higher levels of lysyl oxidase (57). Increases in collagen fibril density as well as immature and mature crosslinks points towards early stages of fibrosis in the MMP antibody-transfer mouse model.

In subsequent experiments, we aimed at reducing the fibrotic activity during anti-mLama3 IgG-induced conjunctival and skin inflammation by the ALDH1 inhibitor disulfiram that had previously been used successfully in a mouse model of scarring allergic eye disease (18). In line with the data from reported by Ahadome et al. we also detected increased mRNA levels of different ALDH isoforms, including *Aldh1b1* in MMP skin, which reinforces the role of the enzyme in fibrotic scarring. Disulfiram was initially approved for management of alcohol dependence and is currently evaluated for treatment of malignancies and fungal infections (58–60). The ALDH1 family was also shown to play a role in atopic dermatitis pathology, though its precise function in the skin and disease mechanisms are not yet understood (61). Furthermore, disulfiram also acts as a copper chelator that was shown to block lysyl oxidase activity, which could potentially also reduce the formation of collagen crosslinks in the extracellular matrix. In the MMP mouse model, systemic treatment with disulfiram led to minor reduction of skin lesions. However, no effects on both oral and conjunctival lesions were apparent following either systemic or local administration of disulfiram. Of note, methodological aspects, were consistent with previously published data and we used the same dose of disulfiram, which was shown to be effective for topical treatment of ocular eye lesions in the allergic eye model (18). Furthermore, systemic treatment was done using a high dose of disulfiram of p.o. 200 mg/kg daily (in comparison the maximum recommended dosage for a 70 kg adult is p.o. 500 mg/day, which corresponds to 7.14 mg disulfiram/kg body weight) (62, 63). Therefore, differences in the animal models and associated immune responses might explain the lack of adequate therapeutic response in experimental MMP.

The allergic eye disease mouse model is an active mouse model where experimental conjunctivitis is induced by immunization of mice with ovalbumin i.p. in combination with the adjuvant aluminum hydroxide as well as pertussis toxin, and subsequent challenge with ovalbumin eye drops (64). This results in an IgE-dependent immune response of the mice. In contrast to IgE-associated ocular allergy, MMP is dominated by IgG and IgA immune responses (1–4). Only a few cases have been published where circulating IgE antibodies were detected in patients with MMP, and to our knowledge only one MMP patient with IgE antibodies against laminin-332 was reported, albeit not in connection with conjunctivitis (65, 66). IgE autoantibodies in pemphigoid disease were mainly reported to target the N-terminal ectodomain of BP180 or BP230 in patients with bullous pemphigoid, a pemphigoid diseases not associated with scar formation and a low rate of mucosal lesions (67–71).

The lack of therapeutic efficacy of disulfiram treatment in experimental MMP suggests different immunopathological pathways as causative for the MMP-associated fibrotic phenotype compared to experimental allergic conjunctivitis. In line with the changes in cross-linking processes in experimental MMP described in the present study, future therapy strategies may use small molecule inhibitors that target pathological collagen crosslinking and restore extracellular matrix homeostasis (72, 73).

The limitations of our study include the selection of different time points chosen to demonstrate the apparent fibrotic changes in experimental MMP vs the evaluation of the therapeutic effect of disulfiram in the MMP mouse model. In context of the systemic approach a later time point, in addition to Day 12, should provide more insight into fibrotic processes in experimental MMP, in particular the chronic/remodeling phase of the disease, in subsequent studies. In addition, the inclusion of a secondary control group to the study design, supplementary to the vehicle and disulfiram-treated groups, would add to the interpretation of the data, particularly in evaluating baseline collagen dynamics.

Taken together, our study revealed new insight in autoantibody-mediated fibrotic scarring demonstrating changes in the collagen architecture with increased fibril density and crosslinking activity, which were particularly prominent in skin lesions in experimental MMP. Lack of treatment response following ALDH inhibition in experimental MMP points to the contribution of other fibrogenic mechanisms to this disease and other IgG/IgA-mediated autoimmune blistering diseases with a propensity for scarring.

## Data Availability Statement

The raw data supporting the conclusions of this article will be made available by the authors, without undue reservation.

## Ethics Statement

The animal study was reviewed and approved by Schleswig-Holstein Ministry of Energy Transition, Agriculture, Environment, Nature and Digitalization (40-3/15, 20-2/17)

## Author Contributions

SP and ES designed the experiments. SP, MP, KB, LD, and LC performed animal experiments and/or histological analyses. HS and JB carried out collagen crosslink analyses. SK performed RNA sequencing and HK as well as AF have undertaken subsequent data and statistical analysis. SP and MP analyzed and interpreted experiments and wrote the manuscript. JB and ES corrected the drafted manuscript. All authors approved the submitted version.

## Funding

The work was supported by the German Research Foundation (SCHM 1686/10-1 to ES), the Schleswig-Holstein Excellence Cluster *Precision Medicine in Chronic Inflammation* (DFG EXC 2167/1, TI-3 to ES), the CRU 303 *Pemphigoid Diseases* (to ES), and a junior grant from the University of Lübeck (J08-2021 to MP).

## Conflict of Interest

The authors declare that the research was conducted in the absence of any commercial or financial relationships that could be construed as a potential conflict of interest.

## Publisher’s Note

All claims expressed in this article are solely those of the authors and do not necessarily represent those of their affiliated organizations, or those of the publisher, the editors and the reviewers. Any product that may be evaluated in this article, or claim that may be made by its manufacturer, is not guaranteed or endorsed by the publisher.
