# Supplementary material for: Increased Fibrosis in a Mouse Model of Anti-Laminin 332 Mucous Membrane Pemphigoid Remains Unaltered by Inhibition of Aldehyde Dehydrogenase
Source: Front Immunol. 2022 Feb 7;12:812627. doi: 10.3389/fimmu.2021.812627 (PMC8858800; doi:10.3389/fimmu.2021.812627)
Supplement: Supplementary file 1 [file Presentation_1.pptx]

## Slide 1
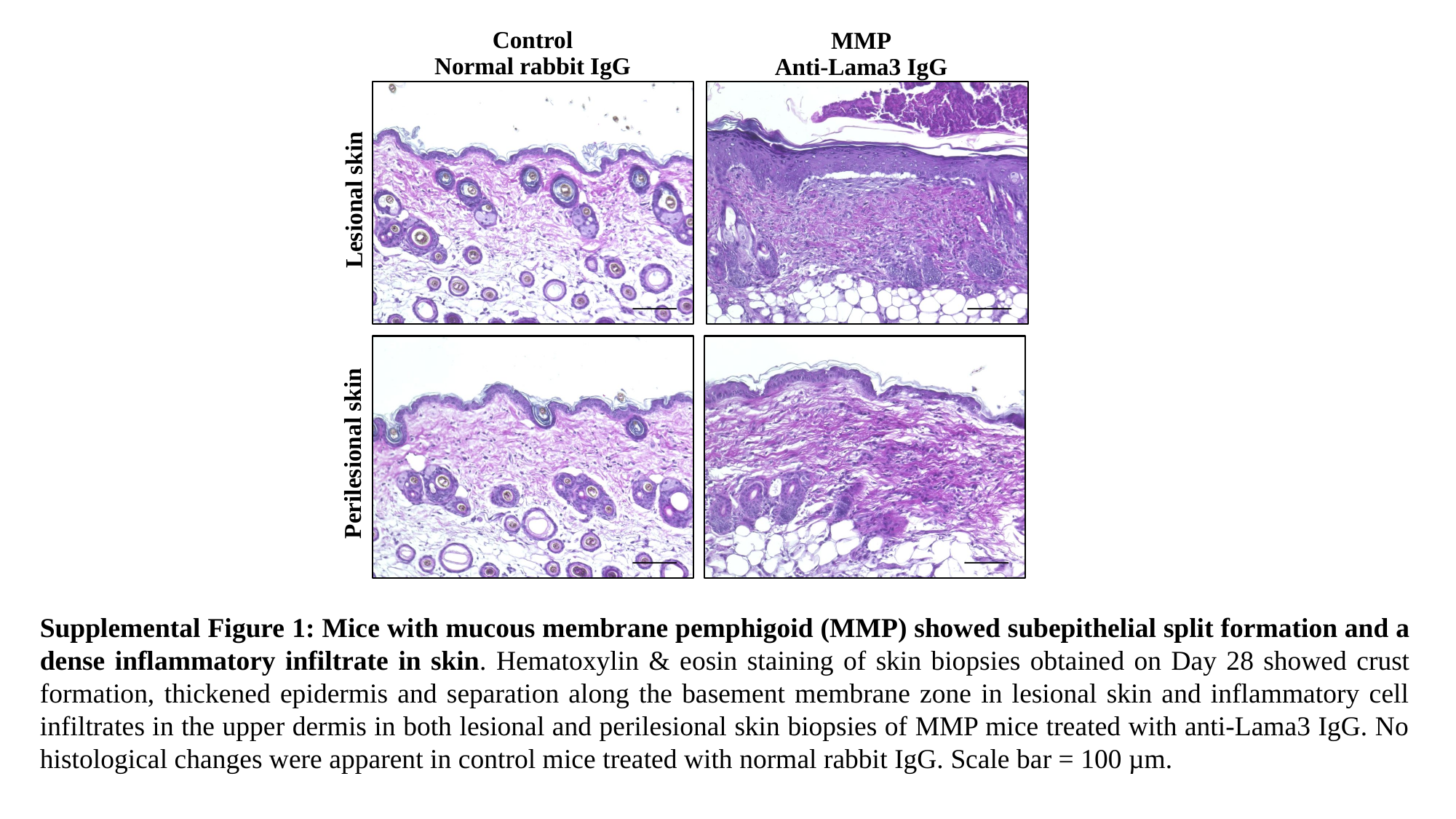

Control
Normal rabbit IgG
MMP
Anti-Lama3 IgG
# Lesional skin
Perilesional skin
Supplemental Figure 1: Mice with mucous membrane pemphigoid (MMP) showed subepithelial split formation and a dense inflammatory infiltrate in skin. Hematoxylin & eosin staining of skin biopsies obtained on Day 28 showed crust formation, thickened epidermis and separation along the basement membrane zone in lesional skin and inflammatory cell infiltrates in the upper dermis in both lesional and perilesional skin biopsies of MMP mice treated with anti-Lama3 IgG. No histological changes were apparent in control mice treated with normal rabbit IgG. Scale bar = 100 µm.

## Slide 2
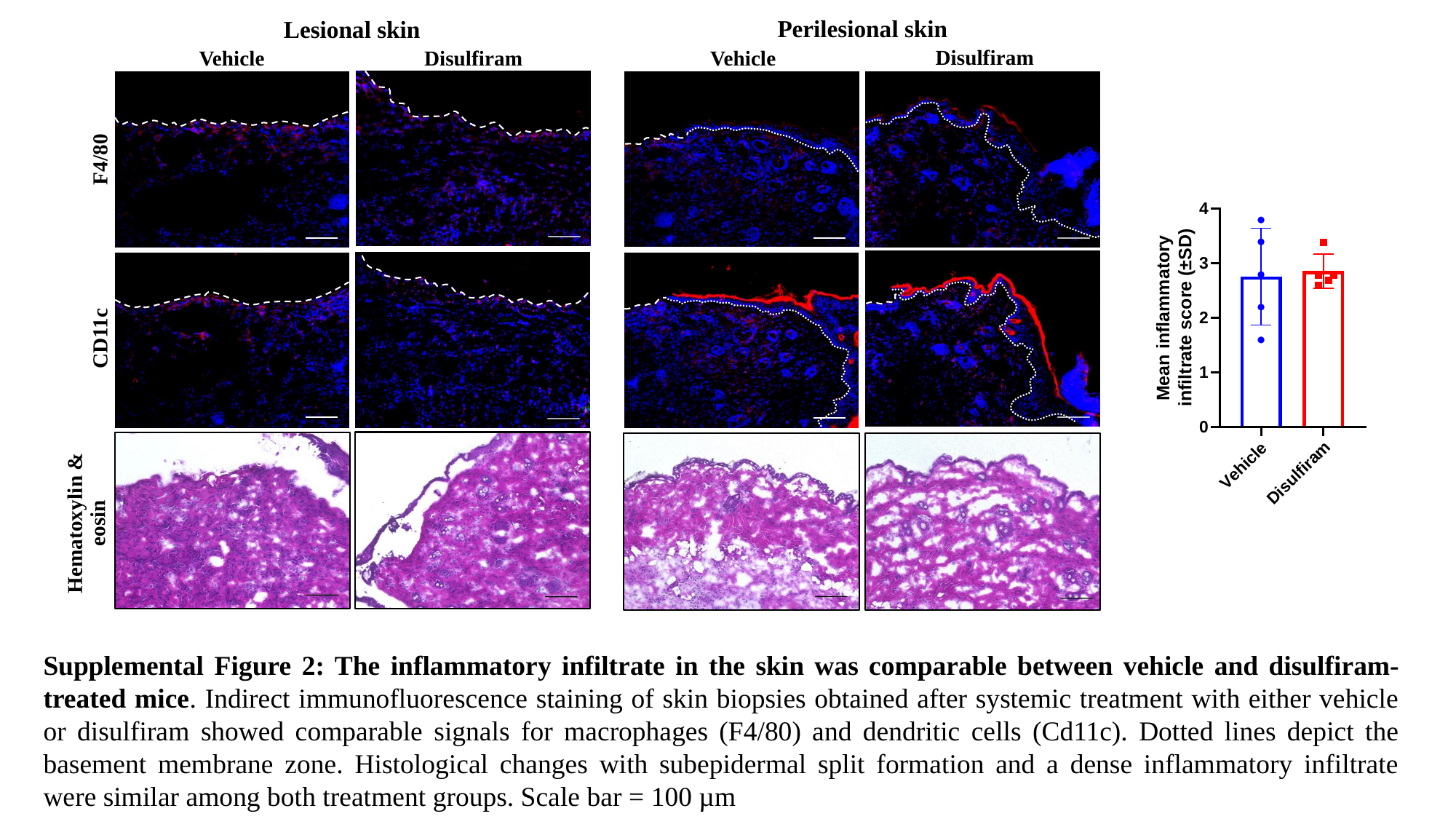

Perilesional skin
Lesional skin
Disulfiram
Vehicle
Disulfiram
Vehicle
F4/80
CD11c
Hematoxylin & eosin
Supplemental Figure 2: The inflammatory infiltrate in the skin was comparable between vehicle and disulfiram-treated mice. Indirect immunofluorescence staining of skin biopsies obtained after systemic treatment with either vehicle or disulfiram showed comparable signals for macrophages (F4/80) and dendritic cells (Cd11c). Dotted lines depict the basement membrane zone. Histological changes with subepidermal split formation and a dense inflammatory infiltrate were similar among both treatment groups. Scale bar = 100 µm
